# The p-STAT3/ANXA2 axis promotes caspase-1-mediated hepatocyte pyroptosis in non-alcoholic steatohepatitis

**DOI:** 10.1186/s12967-022-03692-1

**Published:** 2022-11-02

**Authors:** Yun Feng, Wenhua Li, Zhuoya Wang, Ruling Zhang, Yan Li, Lijuan Zang, Peiwen Wang, Zhenghong Li, Yuwei Dong

**Affiliations:** 1 Department of Gastroenterology, Shanghai General Hospital, Shanghai Jiao Tong University School of Medicine, No.100 Haining Road, Hongkou District, 200080 Shanghai, China; 2 Department of Gastroenterology, Jiading Branch of Shanghai General Hospital, Shanghai JiaoTong University School of Medicine, 800 Huangjiahuayuan Road, 201803 Shanghai, China; 3 Department of Endoscopy Center, The First Hospital of Hunan University of Chinese Medicine, 95 middle Shaoshan Road, Yuhua District, Changsha City, Hunan Province China; 4 Department of Pathology, Shanghai General Hospital, Shanghai Jiao Tong University School of Medicine, 100 Haining Road, Hongkou District, 200080 Shanghai, China; 5 Department of Gastroenterology, Xinhua Hospital, Shanghai Jiao Tong University School of Medicine, No.1665 Konngjiang Road, Hongkou District, 200092 Shanghai, China

**Keywords:** p-STAT3, ANXA2, Caspase-1, Pyroptosis, NASH, Hepatic fibrosis

## Abstract

**Background:**

To explore the roles of Annexin A2 (ANXA2) on hepatocyte pyroptosis and hepatic fibrosis in nonalcoholic steatohepatitis (NASH) and underlying molecular mechanism.

**Methods:**

Bioinformatics analyses were performed on transcriptome data of liver tissues from mice and patients with liver fibrosis for screening the hepatocyte pyroptosis-related differential genes. The in vivo NASH mouse model and in vitro NASH cellular model were established. The expression levels of *Anxa2/*ANXA2 were quantified. Then, the upstream transcription factor of *Anxa2* was screened by ChIP-Seq and experimentally verified. The effects of the p-STAT3/ANXA2 axis on Caspase-1 mediated pyroptosis and fibrosis were explored by in vivo and in vitro experiments.

**Results:**

Bioinformatics analyses suggested that the expression of *Anxa2/*ANXA2 was significantly up-regulated in liver tissues of both NASH mice and patients scoring with high pyroptotic activity. Experimental data showed that the ANXA2 expression was positively associated with the development of hepatocyte pyroptosis and fibrosis. As a transcription factor of ANXA2, p-STAT3 can bind to the promoter of *Anxa2* and promote its transcription. The inhibition of p-STAT3 can significantly suppress hepatocyte pyroptosis and fibrosis, which was significantly reversed after the over-expression of *Anxa2*. Caspase-1 was verified as the player of the p-STAT3/ANXA2 axis to promote pyroptosis and fibrosis. By specifically inhibiting Caspase-1, the promotion effect of the p-STAT3/ANXA2 axis on pyroptosis and fibrosis can be significantly weakened.

**Conclusion:**

The p-STAT3 promoted *Anxa2* expression at the transcription level, thus activating the Caspase-1 mediated hepatocyte pyroptosis and fibrosis in NASH.

**Supplementary Information:**

The online version contains supplementary material available at 10.1186/s12967-022-03692-1.

## Background

Non-alcoholic steatohepatitis (NASH) is a dynamic disease. NASH may regress to steatosis and maintain relatively stable, or it can lead to progressive liver fibrosis, cirrhosis, and other advanced liver diseases [1].

Pyroptosis is a new route of programmed cell death, presenting the characteristics of pro-inflammatory and cytolytic apoptosis, which is mediated by Gasdermin proteins [2]. In the process of pyroptosis, Gasdermin proteins form holes in cell membranes, causing cells to swell and rupture, releasing inflammatory factors to defend against foreign pathogens [3]. This process has been generally reported to depend on the activation of Caspase-1[4, 5], while other studies suggested that the Caspase-1-independent pyroptosis pathway was ubiquitous in mammalian cells, which depended on other activators such as Caspase-4/5/11 [6, 7]. Pyroptosis has been proven to link with several infectious diseases [8], autoimmune disorders [9], and tumors [10].

Hepatocyte pyroptosis has been reported in mice animal models of NASH and human with obesity-associated NAFLD [11, 12]. The hepatocyte pyroptosis was initiated by Caspase-1, which was activated by various inflammasomes. The inflammasomes are intracellular epichaperomes activated in liver cells or other non-platelet cells, which can respond to risk factors. The inflammasomes mainly consist of: [1] pattern recognition receptor (PRR) as sensor molecule, [2] apoptosis-associated speck-like protein containing CARD (ASC), and [3] pro-caspase-1 as effector molecule [13]. As a Caspase-1-dependent programmed cell death, pyroptosis has been proven to make important effects on NASH [14]. However, until now, only limited studies have been performed on hepatocyte pyroptosis in NASH, as well as the molecular mechanism. Therefore, identifying the key molecules involved in the biochemical cascade activating Caspase-1 inflammasomes is significant, which may provide new candidate strategies for developing gene-mediated therapy.

Annexin A2(ANXA2) belongs to the annexin family, which is a calcium-dependent phospholipid-binding protein. ANXA2 is mainly located on the cell membrane. The abnormal expression of ANXA2 has been detected in various malignancies. It has been reported that the expression of ANXA2 was associated with tumorigenesis, progression, invasion, and metastasis, which can be applied as a biomarker for predicting the prognosis [15]. Bioinformatics analysis predicted that ANXA2 was a critical event in steatosis. The deregulation of ANXA2 made effects on fat storage in the liver via deregulating the clearance of plasma cholesterol [16]. The *Anxa2* was also identified as tumor-specific chromatin-accessible regions in hepatocellular carcinoma tissues from NASH [17]. The experimental results showed that ANXA2 expression was associated with not only liver histological features, but also insulin resistance in NASH [18]. The increased expression of ANXA2 in hepatocytes can promote hepatic fibrosis in NASH mice, and the mechanism was to increase the expression of osteopontin. The ANXA2-Notch positive regulatory loop was involved in this process [19]. However, the effects of ANXA2 in NASH-derived hepatocyte pyroptosis have been still unclarified, nor the underlying mechanism.

In this study, the results of both bioinformatic analyses and experiments revealed that, the expression level of ANXA2 was up-regulated in both LPS-treated hepatocytes and NASH liver, and the inhibition of ANXA2 expression can suppress the associated hepatocyte pyroptosis and fibrosis. According to the analysis of upstream transcription factors of ANXA2, it was observed that the highly expressed p-STAT3 could promote the transcription of *Anxa2* by binding with its promoter. The p-STAT3/ANXA2 axis can activate the Caspase-1, thus mediating the hepatocyte pyroptosis and fibrosis of NASH.

## Methods

### Ethics statement

6-week-old C57BL/6J mice were obtained from Charles River Laboratories for constructing the animal model of NASH (Beijing, China) [20, 21]. The mice were maintained in a light/dark cycle of 12 h/12 h, at 22 ± 2 ℃ and 55-65% humidity. All the manipulation relevant to animals have been reviewed and proved by the Animal Ethics Committee of Shanghai General Hospital, and are conducted according to NIH guidelines (Approval No. 2022AW018).

### Bioinformatics analysis

All the public data were obtained from the Gene Expression Omnibus (GEO) database (www.ncbi.nlm.nih.gov/geo). GSE55747 mouse gene chip dataset (including 4 healthy liver tissues and 6 CCL4-induced liver fibrosis tissues) was analyzed for screening differentially expressed genes in the development of liver fibrosis. GSE174478 human gene chip dataset (including 94 patients with non-alcoholic fatty liver disease, NAFLD) was analyzed for screening differentially expressed genes related to hepatocyte pyroptosis. The original data were filtered and standardized by log2(x + 1), and Wilcoxon was used for the difference test. Then, each patient was scored for their pyroptotic activity. The REACTOME_PYROPTOSIS gene set of the Molecular Signatures Database (www.gsea-msigdb.org/gsea/msigdb) was used as the background gene set. The Gene Set Variation Analysis (GSVA) R package was involved in quantifying [22]. Based on the median value, the patients were divided into groups with high and low pyroptotic activity. The ChIP-seq data of STAT3 binding in inflammatory and healthy mouse hepatocytes were downloaded from GSE96767. The obtained Bedgraph format files were converted into BW format files, enrichment heatmaps were drawn for the upstream and downstream 3 kb regions of Transcription Start Site (TSS) using Deeptools, and differential peak enrichment was observed in IGV browser.

### NASH animal model

The NASH mouse model was established according to the previous report [23]. After adapting for one week, the mice were maintained in an animal room with controlled conditions: light/dark cycle of 12 h/12 h, 22 ± 2 ℃, and humidity of 55-65%. The mice in the NASH group were fed with high-fat diet and the mice in the control group were fed a normal diet. The formula of a high-fat diet and a normal diet was shown in Supplementary file S1. All experiments on mice were approved by the Scientific Research Ethics Committee of our hospital (Approval No. 2022AW018). The animals were handled according to the agency’s guidelines for the care and use of experimental animals.

Animals were randomly divided and there were eight [8] mice in each group: Control group (NC group), NASH group, Lv-NC group (NASH mice treated with adenovirus Lv-NC), Lv-sh*Anxa2* group (NASH mice treated with adenovirus Lv-sh*Anxa2*), APTSTAT3-9R group (NASH mice treated with STAT3-specific inhibitor, APTSTAT3-9R), APTSTAT3-9R + Lv-*Anxa2* group (NASH mice treated with both APTSTAT3-9R and Lv-*Anxa2*), Lv-*Anxa2* group (NASH mice treated with adenovirus Lv-*Anxa2*), Lv-*Anxa2* + VX-765 group (NASH mice treated with both adenovirus Lv-*Anxa2* and VX-765). The adenovirus was purchased from Sangon Biotech (Shanghai) Co., Ltd. The mice were injected with a total of 1 × 10^9^ PFU recombinant adenoviruses via tail vein injection. VX-765 was a specific inhibitor for Caspase-1. After experiments, all the mice were euthanized. The serum and liver tissues were collected for the following experiments.

### HE staining

HE staining was applied for detecting pathological changes in the liver. Fresh liver tissue was fixed with 4% paraformaldehyde, embedded in paraffin, and sliced (the thickness was 4 μm). After dewaxing and hydrating, the slices were stained with hematoxylin (Sigma, Shanghai, China) at room temperature (RT), and then rinsed. After differentiating with 5% acetic acid for 1 min, the slices were rinsed and then stained with eosin (Sigma) for 1 min.

### Masson staining

Masson staining was applied for detecting liver fibrosis. The kit was purchased from Solarbio (Beijing, China). After dewaxing and hydrating, the slices were stained with iron hematoxylin for 7 min at 20 ℃, and then rinsed. After differentiating with 1% hydrochloric acid alcohol for 30s, the sections were rinsed and then stained with ponceau acid fuchsin for 5 min. After differentiating the slices with 1% phosphomolybdic acid and 1% acetic acid (each for 1 min), the slices were sealed after dehydration and observed under the microscope.

### Immunohistochemical staining

After dewaxing and hydrating, the antigen of slices was repaired by incubating with 10 mM sodium citrate and 0.05% Tween-20 (pH 7.4) for 10 min at 100 ^o^C. The endogenous peroxidase was blocked with a mixture of H_2_O_2_ (3%): methanol (1:1) for 30 min. After blocking with 5% bovine serum albumin for 1 h at RT, the slices were incubated with α-SMA primary antibody (1: 1000, 14-9760-82, Invitrogen) at 4 ^o^C overnight. The HRP labeled 2nd antibody was added and incubated for 1 h at RT. Images were taken with an upright microscope (Zeiss, Oberkochen, Germany) and analyzed quantitatively by ImageJ according to previous literature [24, 25].

### ELISA

ELISA was applied for quantifying the biomarkers for indicating liver damage and inflammation in mouse serum and hepatocyte culture supernatant, including alanine aminotransferase (ALT), aspartate aminotransferase (AST), and pro-inflammatory factors IL-1β, C-reactive protein (CRP). The ELISA kits were obtained from Abcam (Beijing, China). The immunoassay was performed according to the description of the manufacturer.

### Western blot (WB)

Total protein was extracted with RIPA lysis buffer (Abcam), and then quantified by BCA kit (Abcam). 20 µg of protein was loaded on the SDS-PAGE gel. The protein was transferred to the PVDF membrane after electrophoresis. After blocking, the membrane was incubated with antibodies for relevant biomarkers overnight at 4^o^C, and then incubated with HRP labeled 2nd antibody (1: 10,000, ab205718, Abcam) for 2 h at RT. The immunoreactive stripes were developed with an ECL substrate kit (Abcam). The intensity of stripes was quantified with ImageJ software. The antibodies involved in WB were shown in Table 1.

**Table 1:** Primary antibodies used for WB.

Proteins	Cat.No	Dilution	Manufacturer
ANXA2	03-4400	1:1000	Invitrogen
pro-Caspase1	14-9832-82	1:1000	eBioscience
C-Caspase1	PA5-99390	1:1000	Invitrogen
ASC	PA5-50915	1:1000	Invitrogen
GSDMD	ARG41404	1:1000	Arigobio
GSDMD-N	ab215203	1:1000	Abcam
α-SMA	14-9760-82	1:1000	Invitrogen
p-STAT3	00968	1:1000	Genetex
β-Actin	ab8226	1:1000	Abcam

### RT-qPCR

Total RNA was extracted from liver tissues or hepatocytes and reverse transcribed into cDNA using ReverTra Ace® qPCR RT kit (Toyobo, Japan). RT-qPCR was conducted with a qPCR kit and CFX Connect™ real-time system (BIO-RAD, USA). The relative mRNA expression level of the tested gene was analyzed by 2^−ΔΔCT^, with the internal reference of *Gapdh*. The sequences of primer pairs were provided (Table 2).

**Table 2:** Primer pairs used for RT-qPCR

Gene	Forward primer (5’-3’)	Reverse primer (5’-3’)
*Anxa2*	CACCAACTTCGATGCTGAGAGG	GCACATTGCTGCGGTTTGTCAG
*Gapdh*	CATCACTGCCACCCAGAAGACTG	ATGCCAGTGAGCTTCCCGTTCAG

### ***In vitro*** study on mouse hepatocytes.

Hepatocytes were isolated from 4 mice based on the previous report [26]. The mouse liver was perfused with EGTA buffer (5.4 mM KCl, 0.44 mM KH_2_PO_4_, 140 mM NaCl, 0.34 mM Na_2_HPO_4_, 0.5 mM EGTA, and 25 mM Tricine) to drain the blood. Then, the liver tissue was digested with 0.075% collagenase. All the buffers were perfused at a rate of 5 mL min^− 1^ for 10 min. After digestion, the liver tissue was filtered and centrifuged three times at 750 rpm for 5 min. The cells were cultured in Williams E medium without phenol red, supplemented by primary hepatocyte maintainer, and cultured overnight at 37 ℃ in 5% CO_2_.

The obtained cell suspension was incubated with 0.01% DNase for 15 min, and filtered (pore size: 100 μm). The cells were centrifuged at 200 × g for 10 min for collecting the cell pellet. Hepatocytes were washed with HBSS and cultured in RPMI 1640 medium (Gibco) supplemented with 10% FBS, 100 units mL^− 1^ penicillin, and 100 µg mL^− 1^ streptomycin (Sigma). The hepatocytes were cultured overnight at 37 ^o^C and 5% CO_2_ in 6-well plates coated with collagen. To simulate the hepatocyte under NASH environment, 1 µg mL^− 1^ LPS(Sigma-Aldrich) was applied to treat cells for priming for 5 h, and then BzATP (100 µM) was applied for inflammasome activation for another 1 h.

The sh-*Anxa2* and over-expression DNA plasmid (oe-*Anxa2*) used for cell transfection were purchased from Origene. Lipofectamine2000 was used for transfection. Cells were treated with 10 µM APTSTAT3-9R or VX-765 for 2 h to inhibit the activation of STAT3 or Caspase-1, respectively.

### Luciferase reporter assay

The promoter sequence of *Anxa2* and the potential binding site of p-STAT3 was obtained from the JASPAR database (http://jaspar.genereg.net/). The *Anxa2* promoter sequence integrating this site was inserted in the pGL4-Luc-Report vector (Promega, Shanghai, China) to construct the promoter luciferase reporter. The above vectors were transfected into hepatocytes. Then, the cells were treated with DMSO or STAT3-specific inhibitor APTSTAT3-9R. After 48 h, the luciferase activity was determined by a double luciferase detection kit (Promega).

### ChIP-qPCR

ChIP was performed with Magna ChIP™ A/G Chromatin Immunoprecipitation Kit (Merck Millipore, Burlington, MA, USA) and anti-p-STAT3 (1:50, #9134, CST) antibody or IgG (1:50, #3900, CST). After ChIP, the qPCR was applied for quantifying immunoprecipitated DNA, with the following primers targeted to the *Anxa2* promoter sequence (Forward: CCCAGTTCAGAGGAATCCAA; Reverse: CCAGGCCCTACAAGTATCCA).

### Statistics

All statistics were carried out by Graphpad Prism 8.02 software. The data were expressed as mean value ± standard deviation (SD) for three independent experiments. The difference was compared with the Unpaired *t*-test (two groups), One-way ANOVA, or Two-way ANOVA (multiple groups). Tukey was applied for the post hoc test. *P* < 0.05 indicated statistically significant.

## Results

### The up-regulated expression of ***ANXA2*** in the liver of NASH mice

**Fig. 1:**
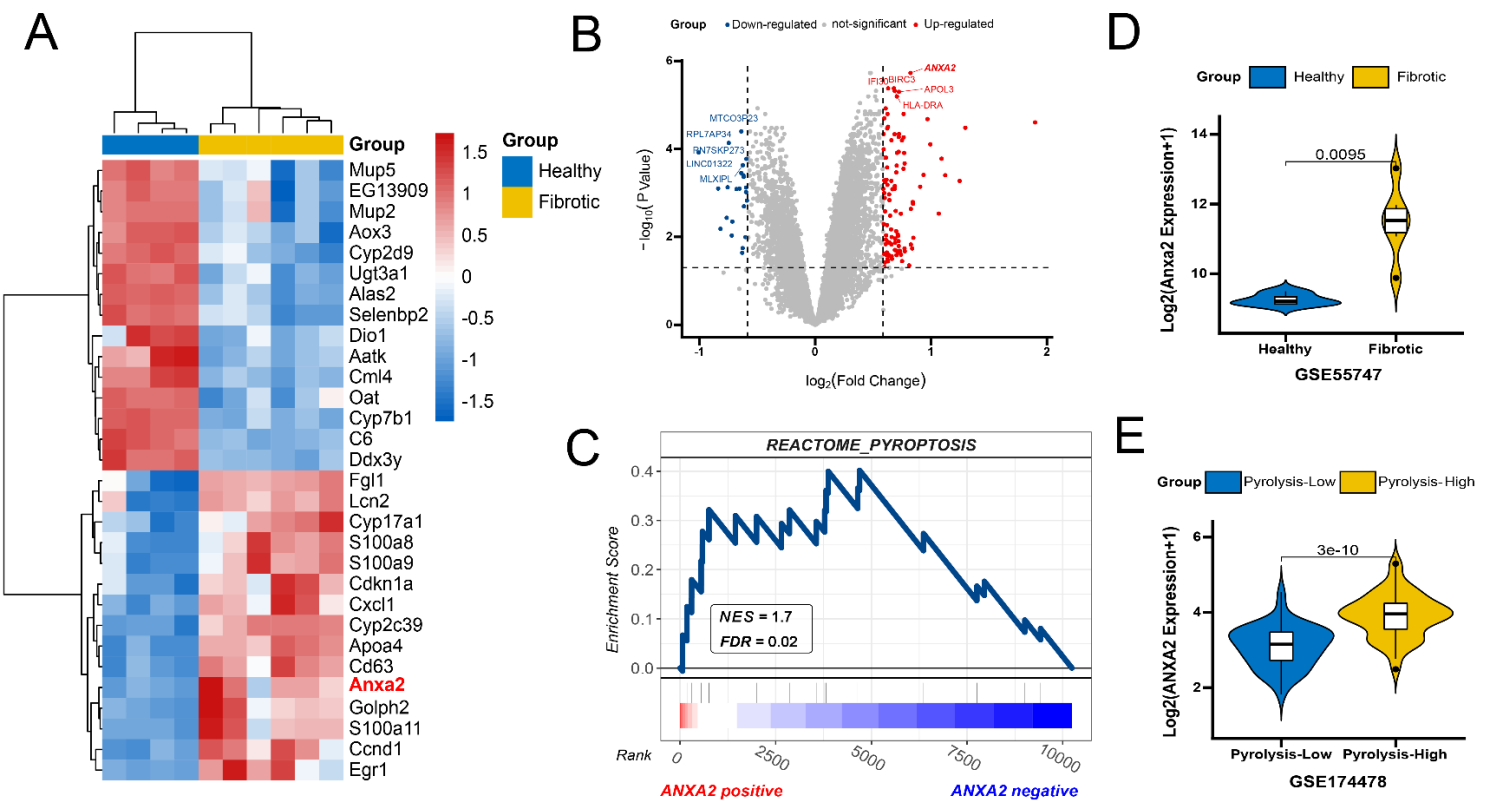
Bioinformatic analysis of elevated expression of *Anxa2*/ANXA2 in NASH. (**A**) Heatmap of differentially expressed genes in GSE55747 dataset. (**B**) Volcano plot of differentially expressed genes in GSE174478 dataset. The red dots are those differential expression gene with log2Foldchange > 0.585 and *P* < 0.05 C. Gene Set Enrichment Analysis of ANXA2 in the NAFLD cohort. NES: normalized enrichment score; FDR: false discovery rate. D. Violin plot for the expression of *Anxa2* in the Fibrotic and healthy tissues of mice. E. Violin plot for the expression of ANXA2 in the pyroptosis-high and pyroptosis -low NAFLD patients, which are divided by the median value of pyroptosis activity score

Activation of the inflammasome in NASH led to pyroptosis of hepatocytes and the development of liver fibrosis [11, 27]. Bioinformatics results indicated that the expression of *Anxa2* was significantly up-regulated in fibrotic tissues of NASH mice (Fig. 1 A). In the NAFLD cohort including 94 patients, a differential analysis was performed on the transcriptome data. The cutoff for screening differential expression gene was set as follows: [1] log_2_Foldchange of 0.585 (corresponding to the expression level changed 1.5 times); [2] *P* < 0.05. The up-regulated expression of ANXA2 was most significant in the high-pyroptosis group (Fig. 1B). The pyroptosis activities were scored with the GSVA R package and separated with the median value (Supplementary file S2). Further, we calculated the Pearson correlation coefficients between ANXA2 and all the other genes in the NAFLD cohort (Supplementary file S3) and performed the GSEA. The result confirmed that the pyroptosis pathway was significantly enriched on the side of ANXA2 positive correlation, indicating that the up-regulated expression of ANXA2 was positively correlated to pyroptosis (Fig. 1 C). The expressions of *Anxa2*/ANXA2 were compared with the Wilcoxon test and shown with violin plots (Fig. 1D and E).

**Fig. 2:**
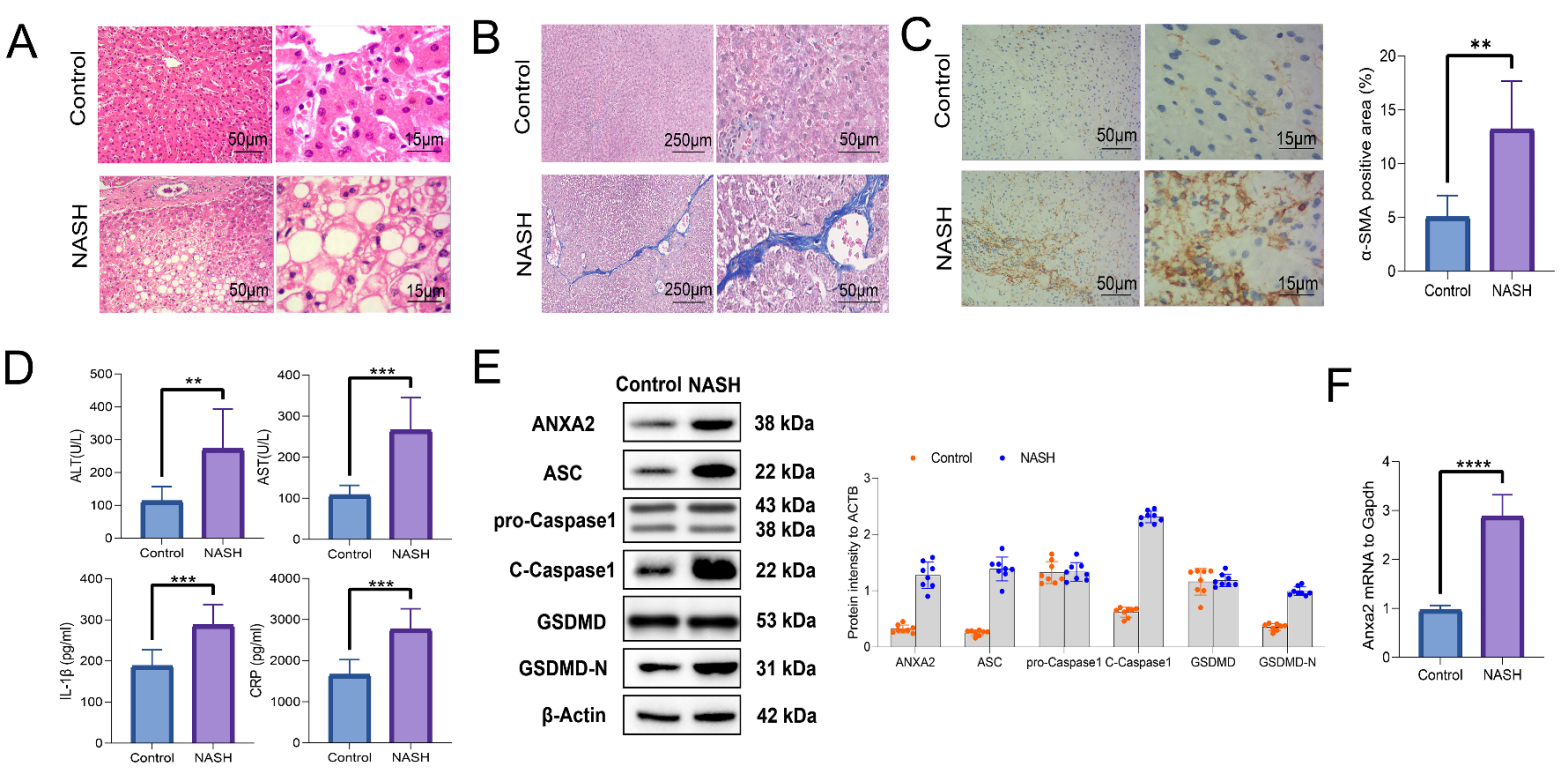
**Elevated expression of ANXA2 contributed to the pyroptosis and fibrosis in NASH.** (**A**) HE staining for indicating pathological changes of liver tissues. (**B**) Masson staining for indicating liver fibrosis. (**C**) Immunohistochemical staining for analyzing the positive rate of α-SMA. (**D**) The serum levels of ALT, AST, IL-1β, and CRP detected with ELISA. (**E**) The expression levels of ANXA2, ASC, pro-Caspase1, C-Caspase1, GSDMD, and GSDMD-N detected with WB. (**F**) The expression of *Anxa2* mRNA detected with RT-qPCR. Eight mice were included in each group. **: *P* < 0.01; ***: *P* < 0.001; ****: *P* < 0.0001

Then, the NASH mouse model was established. The pathological changes were observed. The fat deposition and hepatocyte ballooning can be observed from HE staining of NASH livers (Fig. 2 A). Significant progression of liver fibrosis can be observed in Masson staining (Fig. 2B). It was consistent with the quantitative level of the fibrotic marker α-SMA, which was obtained from immunohistochemical analysis (Fig. 2 C).

The levels of liver injury biomarkers were increased in the serum of NASH mice, including ALT and AST. The pro-inflammatory factor IL-1β and CRP was also significantly elevated (Fig. 2D). The significantly elevated expression of ANXA2 and pyroptosis-related proteins were observed, including ASC, pro-Caspase1, Cleaved-Caspase-1 (C-Caspase1), GSDMD, and GSDMD-N (Fig. 2E). Moreover, the mRNA expression level of *Anxa2* was also significantly elevated (Fig. 2 F).

### **Inhibition of*Anxa2*improved hepatocyte pyroptosis and liver fibrosis in NASH mice**

**Fig. 3:**
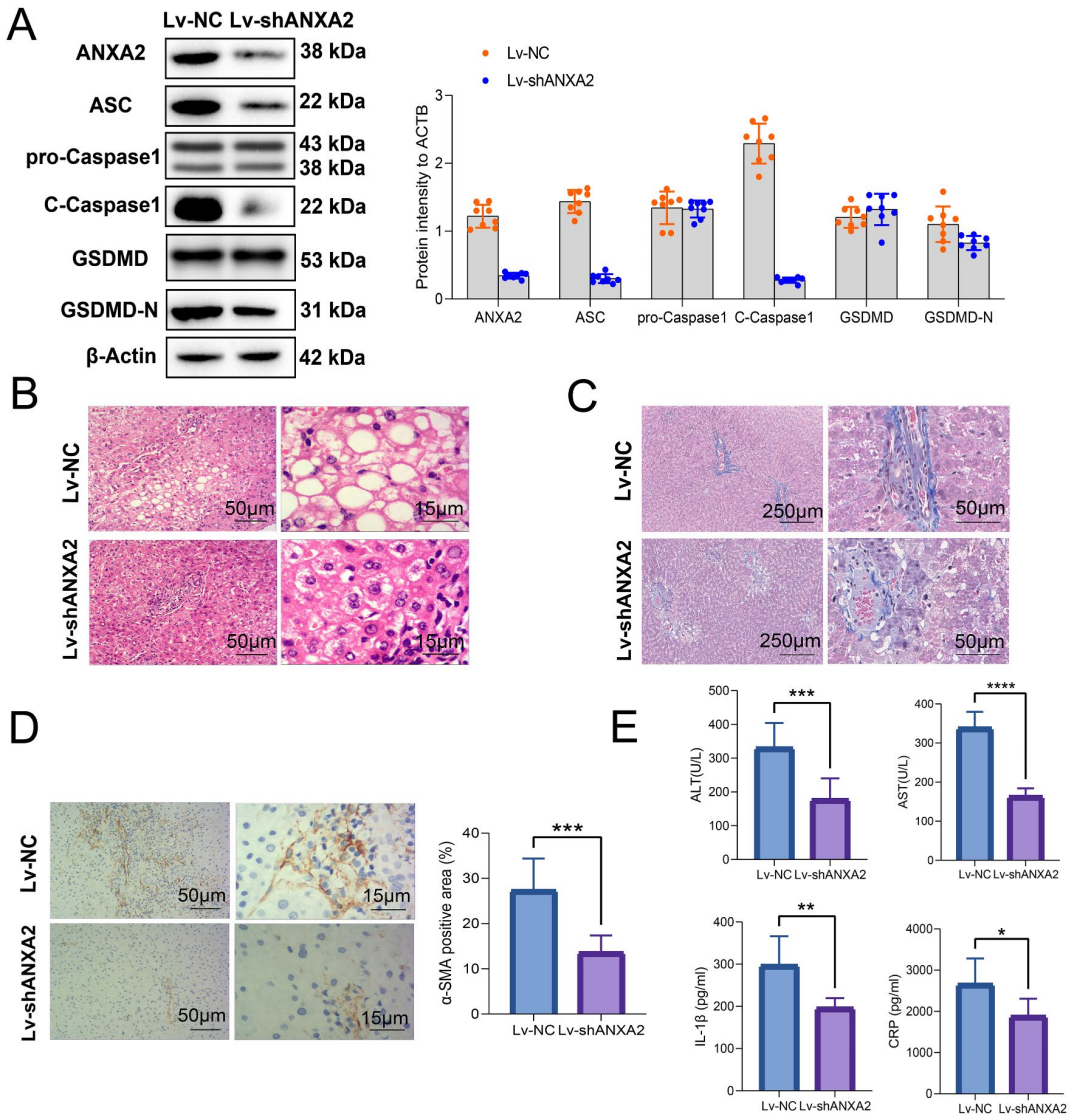
**Inhibition of*Anxa2*improved hepatocyte pyroptosis and liver fibrosis in NASH mice**. (**A**) WB for detecting the inhibitory effects from Lv-sh*Anxa2* on the expression of ANXA2 and pyroptosis related proteins (including ASC, pro-Caspase1, C-Caspase1, GSDMD, and GSDMD-N) in liver tissues of NASH mice. (**B**) HE staining for detecting the pathological changes of NASH liver. (**C**) Masson staining for detecting the effects of *Anxa2* inhibition on liver fibrosis. (**D**) Immunohistochemical staining for detecting the effects from *Anxa2* inhibition on α-SMA expression. (**E**) The serum levels of ALT, AST, IL-1β, and CRP. Eight mice were included in each group. *: *P* < 0.05; **: *P* < 0.01; ***: *P* < 0.001; ****: *P* < 0.0001

NASH mice were treated with adenovirus Lv-sh*Anxa2* for inhibiting the expression of *Anxa2*, with Lv-NC as the control. The expression levels of pyroptosis-related proteins (including ASC, pro-Caspase1, C-Caspase1, GSDMD, and GSDMD-N) in mouse liver tissue were then detected by WB (Fig. 3 A). Lv-sh*Anxa2* significantly suppressed the expression levels of ANXA2, C-CASPASE1, ASC, and GSDMD-N, indicating mitigated pyroptosis.

After the inhibition of *Anxa2* expression, the pathological conditions of the livers of NASH mice can be improved according to the HE staining (Fig. 3B). The liver fibrosis of NASH mice can be significantly improved, revealed by the Masson staining (Fig. 3 C) and α-SMA immunohistochemical staining (Fig. 3D). In addition, the serum levels of ALT, AST, IL-1β, and CRP were also decreased, indicating the relieved hepatocyte injury and inflammation (Fig. 3E).

### **Inhibition of*Anxa2*relieved LPS-induced hepatocyte injury**

**Fig. 4:**
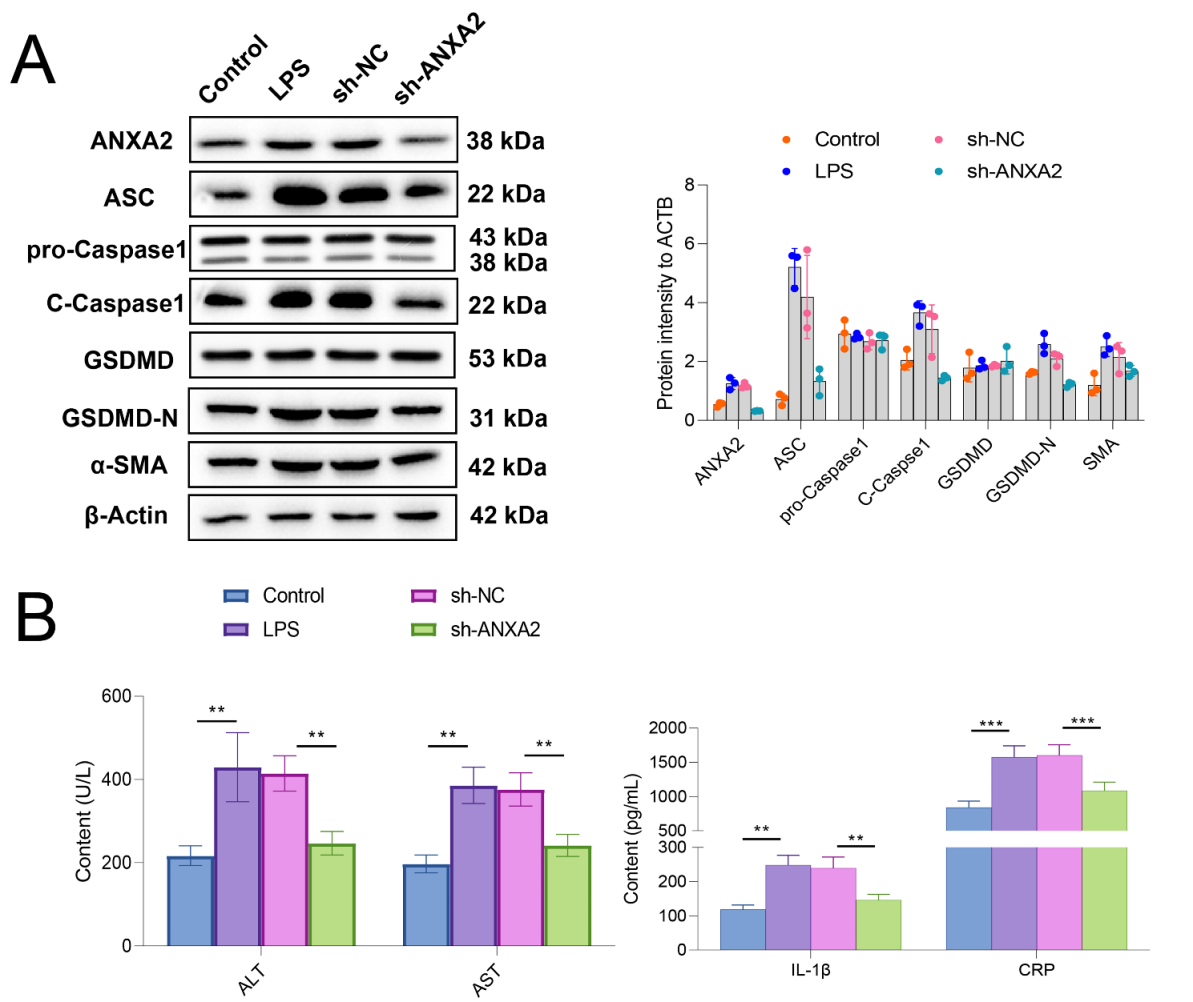
**Inhibition of ANXA2 relieved pyroptosis and fibrosis in LPS-treated hepatocytes.** (**A**) WB for detecting the effects from LPS treatment and sh-*Anxa2* transfection on the expression of ANXA2 and pyroptosis-related proteins (ASC, pro-Caspase1, C-Caspase1, GSDMD, GSDMD-N, and α-SM) in hepatocytes. (**B**) The contents of ALT, AST, IL-1β, and CRP released by the hepatocytes after LPS treatment and sh-*Anxa2* transfection. All cellular experiments were independently repeated three times. **: *P* < 0.01; ***: *P* < 0.001

Hepatocytes were treated with LPS for simulating cell injury. LPS-treated hepatocytes were then transfected with sh-*Anxa2* and corresponding negative control, and pyroptosis-related proteins (including ASC, pro-Caspase1, C-Caspase1, GSDMD, and GSDMD-N) were determined with WB (Fig. 4 A). The results indicated that LPS treatment significantly increased the expression of intracellular ANXA2, while it can be significantly suppressed by inhibition of *Anxa2* with sh-*Anxa2*. At the same time, LPS treatment led to a significantly up-regulated protein expression of C-CASPASE1, ASC, GSDMD-N, and α-SMA, indicating promoted pyroptosis and fibrosis. However, the promotion effect can also be suppressed by the inhibition of *Anxa2*. The supernatant of cell culture for each group was collected and detected. The results showed that LPS treatment significantly induced hepatocytes to release ALT, AST, IL-1β, and CRP, while inhibiting *Anxa2* could significantly relieve the release of the above indicators (Fig. 4B).

### **Promotion of*Anxa2*expression by p-STAT3 at the transcription level**

**Fig. 5:**
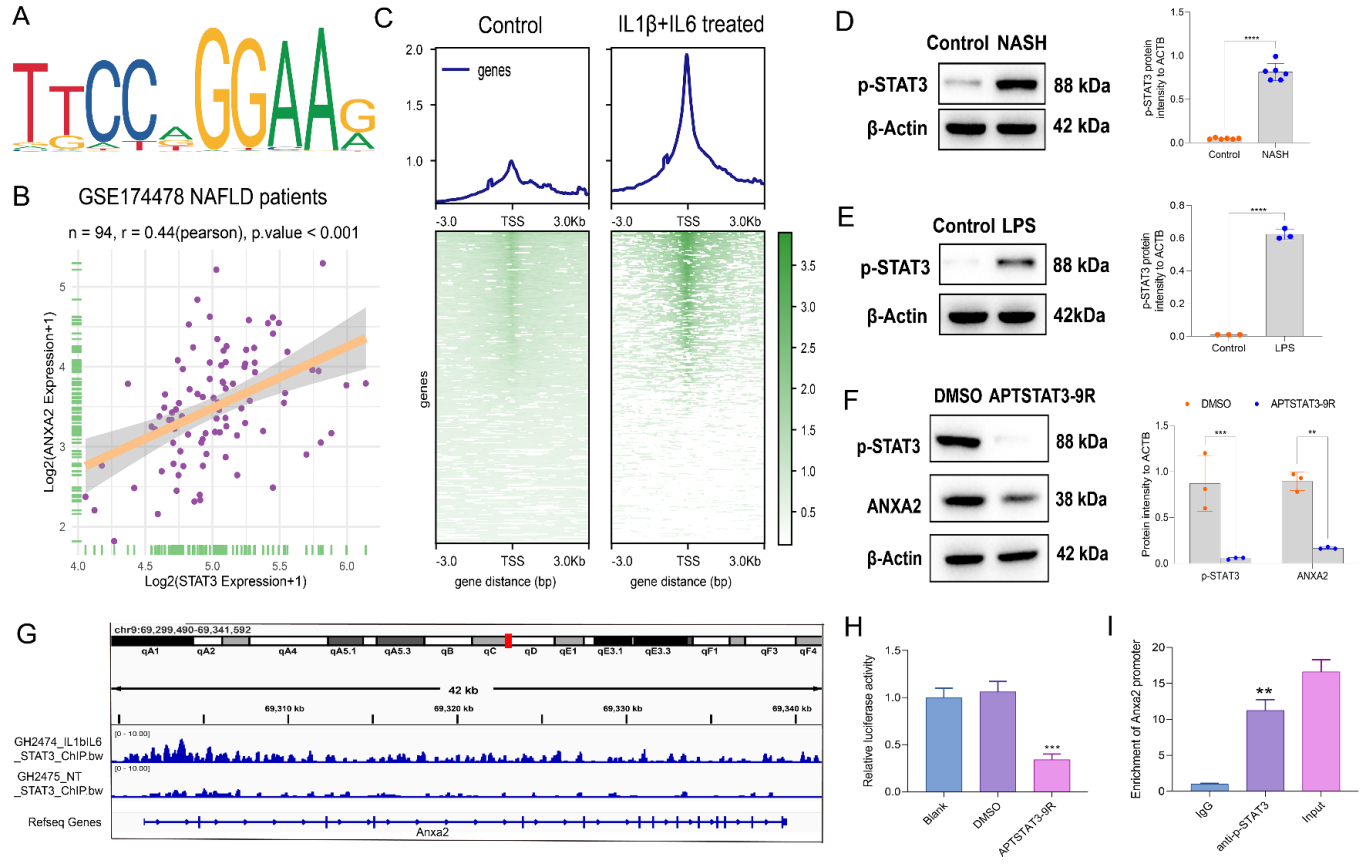
**Promotion of*Anxa2*expression by p-STAT3 at the transcription level.** (**A**) The transcription factor binding motif to *Anxa2* promoter, predicted by JASPAR. (**B**) The Pearson’s correlation plot between STAT3 and ANXA2 in the NAFLD cohort (n = 94). (**C**) Heatmap of ChIP-seq data of STAT3 binding for the upstream and downstream 3 kb regions of Transcription Start Site (TSS) in the pathological and normal states. D-E. WB for detecting the p-STAT3 expression in the liver of NASH mice (**D**) and LPS-treated hepatocytes (**E**). F. WB for detecting the effects from APTSTAT3-9R on p-STAT3 and ANXA2 expression in LPS-induced hepatocytes. G. The enrichment of differential peaks for STAT3 binding in *Anxa2* in inflammatory and normal hepatocytes. H. Promoter luciferase reporter assay for exploring the effects of inhibiting p-STAT3 on the transcriptional activity of *Anxa2*. I. ChIP-qPCR for verifying the binding between p-STAT3 and *Anxa2* promoter. Eight mice were included in each group. All cellular experiments were independently repeated three times. **: *P* < 0.01; ***: *P* < 0.001

The reason for the elevated expression of *Anxa2* in NASH was further explored. The promoter sequence of *Anxa2* was obtained from the UCSC website (Supplementary file S4). The transcription factors (TFs) that can bind to *Anxa2* promoter with relative profile score over 0.95 were predicted at the JASPAR website(https://jaspar.genereg.net) among all the 215 TFs included (Supplementary file S5). On the other hand, the expressions of STAT3 and ANXA2 had the highest co-expression correlation coefficient of 0.44 in the NAFLD cohort. Thus, STAT3 was screened. The motif predicted in the JASPAR database and the Pearson’s correlation plot were shown (Fig. 5 A and 5B). Then, we analyzed the ChIP-seq data of STAT3 binding in inflammatory (IL1β and IL6 treated) and healthy mouse hepatocytes [28]. In the pathological state, the regulation signal of STAT3 to the TSS region of the genome was significantly enhanced (Fig. 5 C).

The WB results revealed that the p-STAT3 expression was significantly up-regulated in the livers of NASH mice (Fig. 5D) and LPS-induced hepatocytes (Fig. 5E). Then, LPS-induced hepatocytes were treated with a specific inhibitor of STAT3 (APTSTAT3-9R). APTSTAT3-9R not only suppressed the phosphorylation of STAT3, but also inhibited the downstream expression of ANXA2 (Fig. 5 F). ChIP-seq data of STAT3 binding showed that the enrichment of differential peaks in *Anxa2* was significantly enhanced in inflammatory hepatocytes (Fig. 5G). To further verify the binding between p-STAT3 and *Anxa2* promoter, the luciferase reporter vector containing this binding site for *the Anxa2* promoter was constructed, and it revealed that the transcriptional activity of the *Anxa2* promoter sequence would be significantly suppressed by the inhibition of STAT3 phosphorylation (Fig. 5 H). The ChIP-qPCR experiment directly demonstrated the binding of p-STAT3 to the *Anxa2* promoter (Fig. 5I).

### The p-STAT3 mediated ***Anxa2***expression for affecting the hepatocyte pyroptosis and liver fibrosis in NASH mice.

**Fig. 6:**
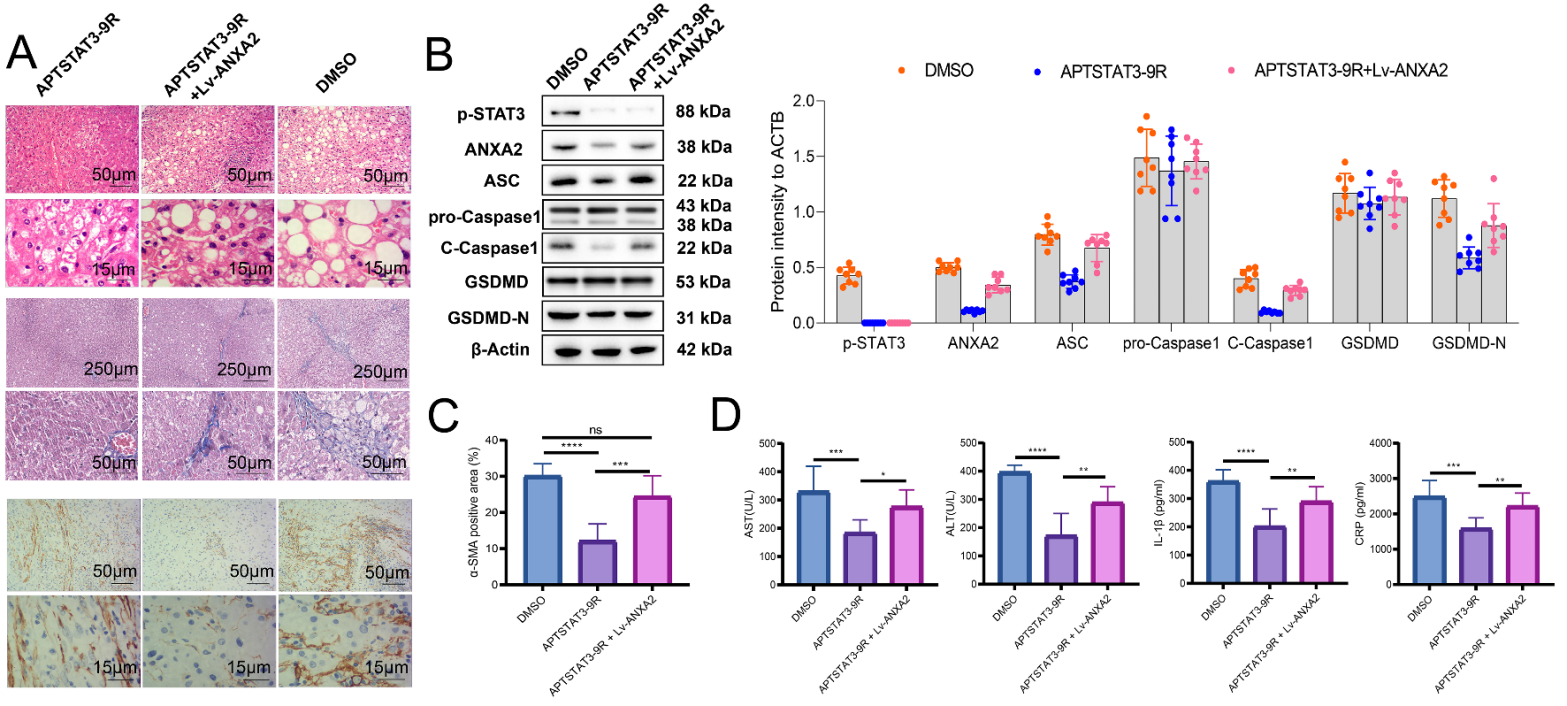
**The over-expression of*Anxa2*reversed the inhibitory effects of p-STAT3 on hepatocyte pyroptosis and fibrosis in NASH mice.** (**A**) HE staining for indicating the pathological changes; Masson staining for detecting liver fibrosis of mice; Immunohistochemical staining for detecting α-SMA expression. (**B**) WB for detecting the expressions of p-STAT3, ANXA2, and pyroptosis-related proteins (ASC, pro-Caspase1, C-Caspase1, GSDMD, and GSDMD-N) in the liver tissues of mice in each group. (**C**) Differentially analysis of the α-SMA positive area. (**D**) The serum levels of ALT, AST, IL-1β, and CRP. Eight mice were included in each group. *: *P* < 0.05; **: *P* < 0.01; ***: *P* < 0.001; ****: *P* < 0.0001

For investigating the mediating role of p-STAT3 on *Anxa2 in vivo*, both the APTSTAT3-9R and over-expressing adenovirus Lv-*Anxa2* were applied to treat NASH mice. From the HE staining results, it was observed that inhibition of p-STAT3 by APTSTAT3-9R significantly improved the liver pathological status of NASH mice, while this improvement can be significantly reversed by the overexpressed *Anxa2* (Fig. 6 A). Meanwhile, p-STAT3 inhibition also mitigated liver fibrosis, indicated by Masson staining and immunohistochemical staining of a-SMA (Fig. 6 A and 6 C). While, fibrosis seemed to be aggravated by the overexpressed *Anxa2* (Fig. 6 A). Related proteins were also detected by WB. APTSTAT3-9R significantly inhibited the expression of p-STAT3, ANXA2, C-CASPASE1, ASC, and GSDMD-N, indicating relieved pyroptosis (Fig. 6B). After the co-treatment of Lv-*Anxa2*, the over-expressing *Anxa2* significantly reversed the expression of the above proteins, but the expression of p-STAT3 was not significantly changed (Fig. 6B).

In addition, the p-STAT3 inhibition reduced the levels of ALT, AST, IL-1β, and CRP in mouse serum, while overexpressed *Anxa2* led to re-elevated levels of related indicators (Fig. 6D).

### The p-STAT3 mediated ***Anxa2***expression for affecting the hepatocyte pyroptosis and fibrosis in LPS-treated hepatocytes.

**Fig. 7:**
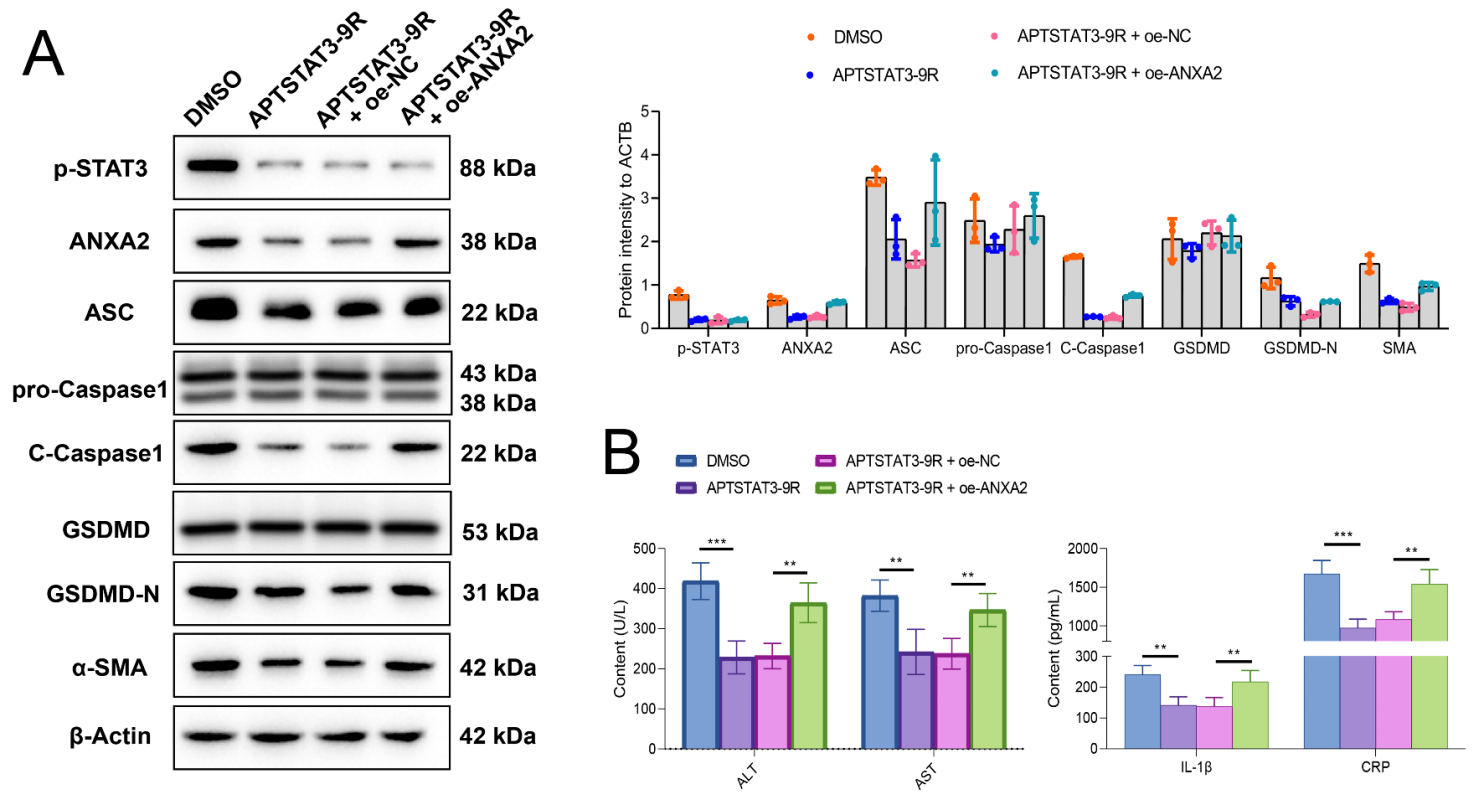
**The over-expression of*Anxa2*reversed the inhibitory effects of p-STAT3 on pyroptosis and fibrosis in LPS-treated hepatocytes.** (**A**) WB for detecting the expressions of p-STAT3, ANXA2, pyroptosis-related proteins (ASC, pro-Caspase1, C-Caspase1, GSDMD, and GSDMD-N) and α-SMA. (**B**) The level of ALT, AST, IL-1β, and CRP in the supernatant of LPS-treated hepatocytes. All cellular experiments were independently repeated three times. **: *P* < 0.01; ***: *P* < 0.001

For further investigating the mediating role of p-STAT3 on *Anxa2*, APTSTAT3-9R (a specific inhibitor of p-STAT3) and oe-*Anxa2* (over-expression plasmid of *Anxa2*) were co-transfected into LPS-treated hepatocytes. The expression of pyroptosis and fibrosis-related proteins (including ASC, pro-Caspase1, C-Caspase1, GSDMD, and GSDMD-N) in the supernatant of hepatocyte culture was determined with WB (Fig. 7 A). APTSTAT3-9R significantly inhibited the expression of these proteins, while after co-transfecting with oe-*Anxa2*, the expressions of other proteins were significantly reversed except for p-STAT3, in which the level of p-STAT3 was not significantly changed. Inhibition of p-STAT3 significantly suppressed the release of ALT, AST, IL-1β, and CRP from hepatocytes, while, the levels of these markers were significantly reversed and up-regulated with overexpressed *Anxa2* (Fig. 7B).

### Caspase-1 is the player of the p-STAT3/ANXA2 axis to promote hepatocyte pyroptosis and liver fibrosis

**Fig. 8:**
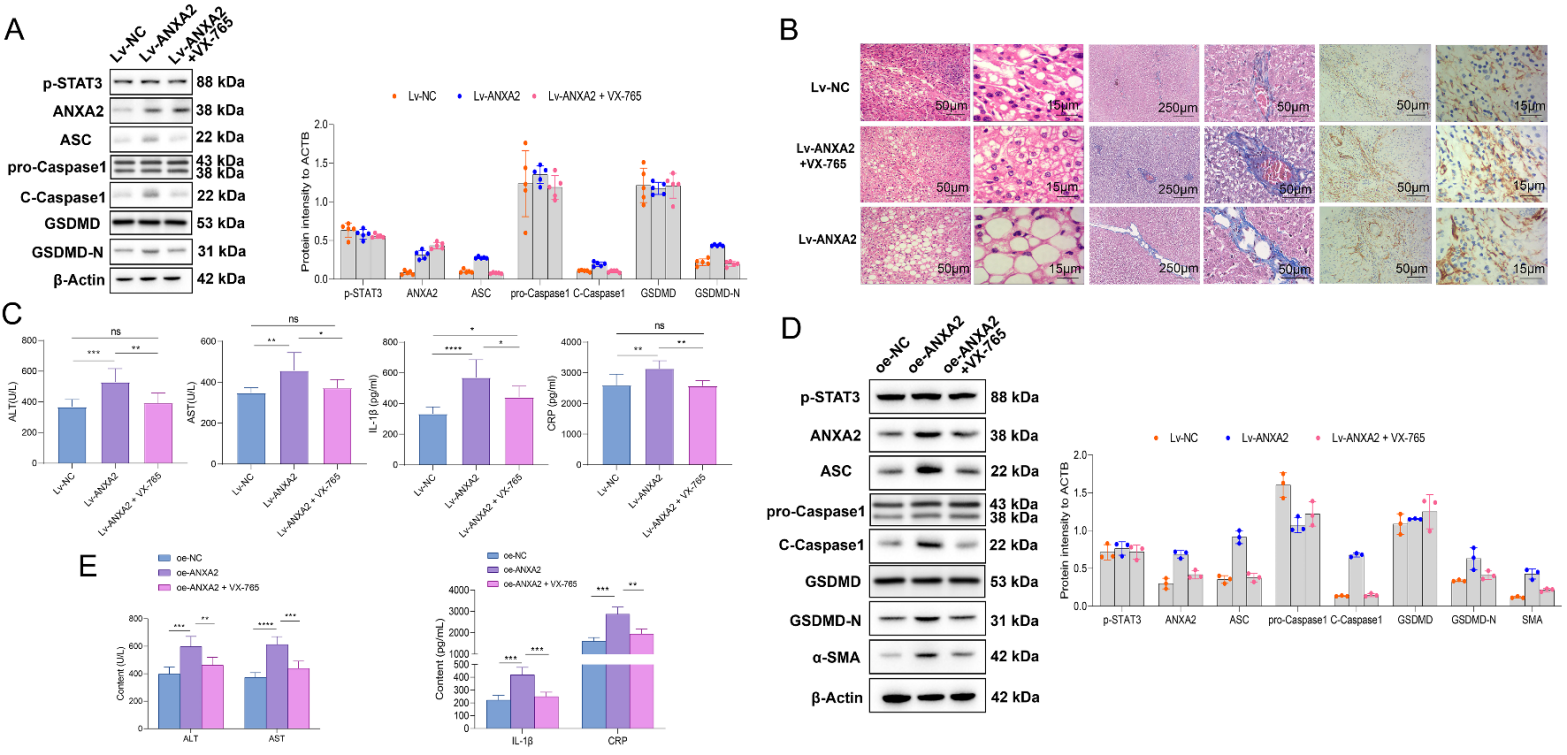
**p-STAT3/ANXA2 axis regulated hepatocyte pyroptosis and liver fibrosis by acting on Caspase-1.** (**A**) WB for detecting the protein expression in the liver tissue of mice in each group, including p-STAT3, ANXA2, pyroptosis-related proteins (ASC, pro-Caspase1, C-Caspase1, GSDMD, and GSDMD-N). (**B**) HE staining for detecting the pathological changes of NASH mouse liver; Masson staining for detecting the liver fibrosis; Immunohistochemical staining for quantifying the α-SMA expression in mouse liver. (**C**) The levels of ALT, AST, IL-1β, and CRP in mouse serum. (**D**) WB for detecting the protein expression in hepatocytes, including p-STAT3, ANXA2, pyroptosis-related proteins (ASC, pro-Caspase1, C-Caspase1, GSDMD, and GSDMD-N), and α-SMA. (**E**) The release levels of ALT, AST, IL-1β, and CRP in hepatocyte supernatant. Five mice were included in each group. All cellular experiments were independently repeated three times. ns: not significant; *: *P* < 0.05; **: *P* < 0.01; ***: *P* < 0.001; ****: *P* < 0.0001

The roles of Caspase-1 were also verified in hepatocyte pyroptosis in NASH. In the in vivo study, the NASH mice were treated with adenovirus Lv-*Anxa2* for overexpressing *Anxa2.* Then, the mice with overexpressed *Anxa2* were administrated with VX-765 (inhibitor for Caspase-1). Related proteins in the liver tissues of mice were detected by WB, including ASC, pro-Caspase1, C-Caspase1, GSDMD, and GSDMD-N (Fig. 8 A). We observed that the over-expression of *Anxa2* upregulated C-CASPASE1 expression, which was accompanied by an increase in the protein expression of ASC and GSDMD-N. However, VX-765 could significantly reverse the promoting effect of Lv-*Anxa2* on the protein expression of C-CASPASE1, ASC, and GSDMD-N, indicating relieved pyroptosis (Fig. 8 A).

In addition, overexpression of *Anxa2* significantly aggravated liver pathological changes in NASH mice, while such a promotion effect could be significantly reversed by the inhibition of Caspase-1 activation with VX-765 (Fig. 8B). Masson and immunohistochemical staining revealed that, overexpression of *Anxa2* aggravated liver fibrosis in NASH mice, and this pro-fibrotic effect was significantly reversed by the inhibition of Caspase-1 (Fig. 8B). Further, the overexpressed *Anxa2* significantly promoted the release of ALT, AST, IL-1β, and CRP in serum, while the promotion effects can also be eliminated by inhibition of Caspase-1 with VX-765 (Fig. 8 C).

In the in vitro study, oe-*Anxa2* was transfected into LPS-treated hepatocytes. Then, the hepatocytes were further treated with VX-765. Protein expression in cells was detected by WB (Fig. 8D). Overexpression of *Anxa2* promoted Caspase-1-mediated pyroptosis and increased the expression of fibrosis-related proteins. After specific inhibition of Caspase-1 by VX-765, the promotion effect of *Anxa2* was significantly reversed (Fig. 8D). Further, the overexpressed *Anxa2* significantly promoted the release of ALT, AST, IL-1β, and CRP in the supernatant, while this promotion effect of p-STAT3 on the release of related factors can be significantly reversed by inhibition of Caspase-1 with VX-765 (Fig. 8E). It suggested the role of Caspase-1 at downstream of the p-STAT3/ANXA2 axis in pyroptosis.

## Discussion

Histopathological changes in NASH include hepatic steatosis and inflammation, with characteristic hepatocyte damage (“ballooning-like” degeneration). NASH has been considered as a potential health threat since it may develop to advanced liver diseases [29]. NASH has become a large and increasing public health problem in worldwide. The growing prevalence may lead to a great disease burden in the future [30]. Fortunately, NASH is reversible, which can be timely controlled. Several studies have attempted to investigate the molecular mechanism of NASH and develop potential therapeutic strategies [31]. Here, we provide in vivo and in vitro evidence that *Anxa2* promotes hepatocyte pyroptosis and liver fibrosis. The mechanism is that p-STAT3/ANXA2 axis promotes the activation of downstream Caspase-1, thus inducing hepatocyte pyroptosis in NASH. Importantly, inhibition of the p-STAT3/ANXA2 axis or activation of Caspase-1 has therapeutic implications in the NASH mouse model.

Pyroptosis is a newly characterized type of programmed cell death accompanied by inflammation. Its functions in the development and progression of various diseases remain to be explored. Several studies have tried to apply bioinformatics analysis to explore pyroptosis-related genes, especially in various cancers. One study has compiled 43 pyroptosis-related genes, depicted their expression changes across 31 cancer types, and constructed a novel prognostic risk model to predict cancer patient survival [32]. In another study on lung adenocarcinoma patients, the pyroptosis-related genes with differential expressions were screened from the RNA sequencing and clinical information datasets. From a total of 33 pyroptosis-related genes, 10 key genes were selected by a machine learning algorithm, assisted in subtyping the patients and predicting the overall survival [33]. Another comprehensive bioinformatics analysis also performed on lung adenocarcinoma identified a prognostic pyroptosis-related gene signature containing five genes (NLRP7, NLRP1, NLRP2, NOD1, and CASP6). In addition, a lncRNA KCNQ1OT1/miR-335-5p/NLRP1/NLRP7 regulatory axis was predicted based on the ceRNA network [34]. In a study on breast cancer, “PyroptosisScore” was calculated based on the expression of pyroptosis-related genes. It was generated to quantitatively evaluate pyroptosis in individual patients, which can help clinicians to understand the characteristics of tumor microenvironment infiltration and assist in developing individual immunotherapy [9]. Some studies have also screened the pyroptosis-related genes associated with liver diseases, mainly liver cancer, such as its diagnosis, prognosis [35], immune infiltration [36], immune activity [37], and so on. Seven pyroptosis-related genes (BAK1, BAX, CHMP2A, CHMP4C, CHMP6, GSDMC, and GSDMD) were identified as diagnostic markers, while another four (TP53, GPX4, GSDMC, BAK1) suggested prognostic prediction significance [35]. Besides screening markers, other studies have also tried to find the core gene as well as the potential regulatory axis. CASP8 was identified as the core gene in predicting the prognosis of patients with liver cancer. Moreover, the lncRNA MIR17HG/hsa-miRNA-130b-3p/CASP8 regulatory axis was revealed [38].

Besides various cancers, studies have shown that hepatocyte pyroptosis plays a role in NASH. In a NASH mouse model induced by feeding cholesterol, Sphingomyelin synthase 1 (SMS1) was reported to mediate hepatocyte pyroptosis, thereby inducing NASH [39]. SMS1 was an enzyme that links ceramide to sphingomyelin synthesis and diacylglycerol generation. Higher levels of free cholesterol in hepatocytes induced an increased level of *Sms1.* Under the action of elevated level of SMS1, more diacylglycerol was generated, thus activating KCδ and initiating the DAG-PKCδ-NLRC4 axis. The downstream NLRC4 inflammasome was then activated, thus inducing hepatocyte pyroptosis. In another study performed on alcoholic hepatitis (AH), a Caspase-11-dependent pyroptosis pathway has been performed. GSDMD was activated in AH livers in mice and patients and then inducted pyroptosis downstream of CASP11/4 activation [40]. The arsenic-induced pyroptosis in NASH involved autophagy, CTSB, and the NLRP3 inflammasome cascade, and that taurine alleviated As_2_O_3_-induced liver inflammation by inhibiting the autophagic-CTSB-NLRP3 inflammasomal pathway rather than decreasing lipid accumulation [41]. In a similar study on NAFLD, *Ecklonia cava* extracts or dieckol attenuated NAFLD by decreasing the NLRP3 inflammasome and pyroptosis [42]. The gap junctions mediate intercellular communication and support liver homeostasis, and the connexin hemichannels were preferentially opened by various pathological stimuli, including inflammation and oxidative stress. The latter are essential features of non-alcoholic steatohepatitis. The involvement of connexin32 and connexin43 hemichannels suggested their role as potential drug targets in NASH [43]. As a major building block of hepatocellular gap junctions, connexin32 was involved in the sequelae of steatosis, which underlined progression of NASH [44]. However, the experimental evidence for exploring the mechanism of pyroptosis has been limited.

Other studies have demonstrated the mechanism of NASH. The oxidative stress has been considered a common pathogenetic factor [45]. The hepatotoxicity induced by bupropion resulted from the generated reactive oxygen species (ROS), which can be mitigated by drugs with reactive radical scavenging properties [46]. Some components, such as Silymarin, a standardized extract of the *Silybum marianum*, is believed to make hepatoprotective effects through inhibition of free radicals and inflammation [47]. The oxidative stress and inflammation have been commonly investigated in the mechanism of NASH.

Different from most of the above studies, our study has been conducted from the molecular level, the role of ANXA2 in pyroptosis and fibrosis in NASH has been investigated from both in vivo and in vitro evidence. In this study, we first involved the GEO database for identifying *ANXA2* as a liver fibrosis-related gene, which has so far been rarely discussed. Alcoholic liver disease, alcoholic fatty liver disease, and NASH have all been implicated in the progression of fibrosis. After confirming the upregulation of ANXA2 in liver tissues of NASH mice, the loss-of-function experiment was performed with adenoviral vectors. Knockdown of *Anxa2* significantly inhibited hepatocyte pyroptosis (NLRP3, C-CASPASE1, GSDMD-N), fibrosis (α-SMA), and inflammation (IL-1β, CRP). The inflammatory role of the p-STAT3/ANXA2 axis has been innovatively revealed, which exerted its role through the activation of the NLRP3/caspase-1 inflammasome, thus inducing the pyroptosis.

ANXA2 was an important member of the annexin protein family. It exhibited high-affinity binding for calcium ion and phospholipids [15]. Aberrant expression of ANXA2 was associated with the development of many cancers [39]. *Anxa2* was significantly elevated in both NASH mouse liver and LPS-treated hepatocytes. Meanwhile, inhibiting the overexpression of *Anxa2* can significantly improve hepatocyte pyroptosis and liver fibrosis, either in NASH mice in vivo or LPS-induced hepatocytes in vitro. This suggests a causal link between *Anxa2* and hepatocyte pyroptosis in NASH, at the transcriptional level. In addition, the loss of function experiments can also be applied for screening specific drugs. Some known drugs have been applied for protecting against cell pyroptosis. Metformin protects against intestinal ischemia-reperfusion injury in a TXNIP-NLRP3-GSDMD-dependent manner [48]. The nature compound Wedelolactone with strong anti-inflammatory and antioxidant activities, which could protect against acute pancreatitis and relevant lung injury against pyroptosis and ferroptosis [49]. The methane offered a protective effect for septic mice via its anti-inflammation, anti-oxidation, anti-pyroptosis, and anti-apoptosis properties [50].

To explore the upstream regulators of ANXA2, we used synthetic bioinformatics tools to identify p-STAT3 as a transcription factor responsible for ANXA2 upregulation in NASH. Mice with overexpressed p-STAT3 have been described as a model of systemic sclerosis associated with organ fibrosis [51]. Nonetheless, the pro-inflammatory effects of p-STAT3 have been validated under different conditions [52, 53]. Studies have shown that IL-6 played a clear role in liver deterioration and tumor progression, directly affecting the survival of hepatocellular carcinoma patients. Overexpressed IL-6 level was positively correlated with poor liver function, tumor progression, clinical severity, and 6-month mortality. The mechanism of IL-6 biological activity was mainly through the activation of tissue p-STAT3 [54]. The p-STAT3 has also been a key protein related to epithelial-mesenchymal transition (EMT) in hepatoma cells, and its elevated level indicated the tendency of EMT in inflammatory hepatocytes [55]. The members of the TNF protein superfamily were among the best-characterized inducers of hepatocyte cell death, a hallmark of liver injury [56]. The pro-inflammatory cytokine TNF-α was another crucial factor for the activation of caspase-1-dependent hepatocyte pyroptosis in patients with obesity and NASH [12]. In our study, p-STAT3 promoted the expression of *Anxa2* at the transcription level via binding with the promoter of *Anxa2*. The specific inhibition of p-STAT3 can significantly reduce the expression of ANXA2, C-CASPASE1, ASC, and GSDMD-N, thereby improving hepatocyte pyroptosis and fibrosis in NASH. Meanwhile, overexpression of *Anxa2* significantly reversed the effect of inhibiting p-STAT3. Caspase-1 acted as the player of the p-STAT3/ANXA2 axis to promote hepatocyte pyroptosis and fibrosis, while the inhibition of its activity can significantly reduce the pro-fibrosis effect.

## Conclusion

Both in vivo and in vitro evidence revealed the significant role of ANXA2 in pyroptosis and fibrosis in NASH. The inflammatory role of the p-STAT3/ANXA2 axis has been innovatively demonstrated for the first time. We confirmed that the p-STAT3/ANXA2 axis exerted its role through the activation of the NLRP3/caspase-1 inflammasome, thus inducing pyroptosis in NASH. By specific inhibition of p-STAT3 activation, pyroptosis and fibrosis can be improved in NASH.

## Electronic supplementary material

Below is the link to the electronic supplementary material.

Supplementary Material 1

Supplementary Material 2

Supplementary Material 3

Supplementary Material 4

Supplementary Material 5

Supplementary Material 6

## Data Availability

The datasets used and/or analyzed during the current study available from the corresponding author upon reasonable request.

## Abbreviations

NASH: Non-alcoholic steatohepatitis
ANXA2: Annexin A2
NAFLD: non-alcoholic fatty liver disease
GEO: Gene Expression Omnibus
GSVA: Gene Set Variation Analysis
GSEA: Gene Set Enrichment Analysis
