# Supplementary material for: The p-STAT3/ANXA2 axis promotes caspase-1-mediated hepatocyte pyroptosis in non-alcoholic steatohepatitis
Source: J Transl Med. 2022 Nov 2;20:497. doi: 10.1186/s12967-022-03692-1 (PMC9632054; doi:10.1186/s12967-022-03692-1)
Supplement: Supplementary file 1 — Supplementary Material 1 [file 12967_2022_3692_MOESM1_ESM.docx]

Supplementary Table 4. Formula of standard diet and HFD

| Formula (g/kg) | Standard Diet | High-Fat Diet |
| --- | --- | --- |
| Casein | 195 | 195 |
| DL-Methionine | 3 | 3 |
| Sucrose | 191.1 | 191.1 |
| Dextrose, anhydrous | 66.45 | 64.45 |
| Fructose | 66.45 | 64.45 |
| Corn Starch | 235.03 | 167.43 |
| Maltodextrin | 100 | 0 |
| Anhydrous Milkfat | 30 | 210 |
| Soybean oil | 10 | 0 |
| Cholesterol | 0 | 1.5 |
| Cellulose | 50 | 50 |
| Mineral Mix, AIN-76 (170,915) | 35 | 35 |
| Potassium Citrate, monohydrate | 4.03 | 4.03 |
| Calcium Carbonate | 4 | 4 |
| Vitamin Mix, Teklad (40,060) | 10 | 10 |
| Ethoxyquin, antioxidant | 0.04 | 0.04 |
