# Supplementary material for: The p-STAT3/ANXA2 axis promotes caspase-1-mediated hepatocyte pyroptosis in non-alcoholic steatohepatitis
Source: J Transl Med. 2022 Nov 2;20:497. doi: 10.1186/s12967-022-03692-1 (PMC9632054; doi:10.1186/s12967-022-03692-1)
Supplement: Supplementary file 4 — Supplementary Material 4 [file 12967_2022_3692_MOESM4_ESM.docx]

>mm39_knownGene_ENSMUST00000034756.15 range=chr9:69359902-69398787 5'pad=0 3'pad=0 strand=+ repeatMasking=none

tacgagtgaaccacaagcacacacaactagatggaagagaatcgttagaa

cctagagaaagcagagaggtagactctgcaaggacggagttgaaagacct

agtgtgactgggcagcggtggtggtgtacacttttaattccagcactggg

gaggcagaggcaggcagatctctgattccagacgagcctagtctccagac

aaccagatctatacagagaaaccctgccttgaaaaacaaaaacaaaacaa

aacaaaacaaaacaaaagaaccaaaactagtgtttaaggtatttccagct

ctcttgctagcttgaagtttaatgcggtgtgaacagtcacccacaaacac

agttcactactgcgttgtttcctggaaaagcaagctagaaaagaaacaat

atgacccagagaaacagtttgtcaaggaatgagaagggggaaaaaccttc

aataacctacaattaggaaaagaccgtaggacctggggtgtattggagta

ctgggggaaggaggtaccttctagccttacctcttagtctccctagctgg

ttagaggcctctccgctttgatttacaaggcggagtagactgtgtaaagc

agggccaagcgaacaccctcctcacactgctccagctggtgaccagaggt

taagctaatatgtttgcagacagtgatggagagctgggtgccaacggccc

agttcagaggaatccaaatatatgcgccttgaaacaattctaccctccat

tacctcgctgcctagaagccttttaaaagcaaaagaagtgagttgggcaa

gccaagcagctgaaatcttaataacccagagccagcctgagcagggccct

gtactctaggagtaaggggatttgggcgggggaaatgggtggatacttgt

agggcctggggaggcggtggagctgtcacgtagggcaggactgggcttgg

gctacgttccaagttggatgagtcacccagcagggtgggaacaggccggg
