# Supplementary material for: The p-STAT3/ANXA2 axis promotes caspase-1-mediated hepatocyte pyroptosis in non-alcoholic steatohepatitis
Source: J Transl Med. 2022 Nov 2;20:497. doi: 10.1186/s12967-022-03692-1 (PMC9632054; doi:10.1186/s12967-022-03692-1)
Supplement: Supplementary file 6 — Supplementary Material 6 [file 12967_2022_3692_MOESM6_ESM.pdf]

Shanghai General Hospital,
Shanghai Jiao Tong University School of Medicine

Department of Gastroenterology, Shanghai 200080, China.
Tel: +86-021-63240090, Fax: +86-021-63240825
Email: yuwei.dong@shgh.cn

Yuwei Dong, M.D, Ph.D.
Surgeon, Medical Researcher
Professor of Gastroenterology
June 14, 2022

June 14, 2022

Jounral of Translational Medicine

Dear Editor,

On behalf of all authors, I am delighted to submit a manuscript entitled "**The p-STAT3/ ANXA2 axis promotes Caspase-1-mediated hepatocyte pyroptosis in non-alcoholic steatohepatitis**" to your appreciation and to the editorial analysis of the Jounral of Translational Medicine, as to the possibility of its publication.

In our study, the results of both bioinformatic analyses and experiments revealed the expression level of ANXA2 was up-regulated in both LPS-treated hepatocytes and NASH liver, and the inhibition of ANXA2 expression can suppress the associated hepatocyte pyroptosis and fibrosis. According to the analysis of upstream transcription factors of ANXA2, it was observed that the highly expressed p-STAT3 could promote the transcription of Anxa2 by binding with its promoter. The p-STAT3/ANXA2 axis can activate the Caspase-1 and thus mediating the hepatocyte pyroptosis and fibrosis of NASH. Our study is the first to report the role of p-STAT3/ANXA2 axis in NASH, providing a new therapeutic target for clinical treatment.

This manuscript has not been published before and it is not being considered for publication elsewhere. All the authors have contributed to this manuscript for important intellectual content and read and approved the final manuscript. We declare there is no conflict of interest.

Many thanks for your time and consideration. We deeply appreciate your consideration of our manuscript, and we look forward to your decision. If you have any queries, please don't hesitate to contact me.

Yours sincerely,

Yuwei Dong, M.D, Ph.D.

Department of Gastroenterology,

Shanghai General Hospital

Shanghai Jiao Tong University School of Medicine

Email: yuwei.dong@shgh.cn
